# Cluster of Legionellosis Cases Associated with Manufacturing Process, South Carolina, USA, 2022

**DOI:** 10.3201/eid3101.240916

**Published:** 2025-01

**Authors:** Hani M. Mohamed, Lindsay Zielinski, Abdoulaye Diedhiou, Nakia Clemmons, Jessica C. Smith, Jessica L. Rinsky, Troy Ritter, Melisa Willby, Nancy Burton, Karl Feldmann, Kevin Dunn, Rebecca Whisenhunt, Victoria Greer, Alberto M. Acosta, Mitchell Garber, Claressa E. Lucas, Kelley C. Henderson, Chris Edens, Linda Bell

**Affiliations:** South Carolina Department of Health and Environmental Control, Columbia, South Carolina, USA (H.M. Mohamed, A. Diedhiou, R. Whisenhunt, V. Greer, L. Bell); Centers for Disease Control and Prevention, Atlanta, Georgia, USA (L. Zielinski, N. Clemmons, J.C. Smith, T. Ritter, M. Willby, C.E. Lucas, K.C Henderson, C. Edens); National Institute for Occupational Safety and Health, Cincinnati, Ohio, USA (J.L. Rinsky, N. Burton, K. Feldmann, K. Dunn); Weill Cornell College of Medicine, New York, New York, USA (A.M. Acosta); Engineering Systems Inc., Aurora, Illinois, USA (M. Garber)

**Keywords:** legionellosis, *Legionella pneumophila*, manufacturing facilities, water, occupational health, bacteria, respiratory infections, South Carolina, United States, Legionnaires’ disease

## Abstract

Evolving technology and the development of new devices that can aerosolize water present a risk for new sources of *Legionella* bacteria growth and spread within industrial settings. We investigated a cluster of legionellosis among employees of a manufacturing facility in South Carolina, USA, and found 2 unique equipment sources of *Legionella* bacteria. The cluster of cases took place during August–November 2022; a total of 34 cases of legionellosis, including 15 hospitalizations and 2 deaths, were reported. *Legionella pneumophila* was isolated from 3 devices: 2 water jet cutters and 1 floor scrubber. *L. pneumophila* sequence type 36 was identified in environmental isolates and 1 patient specimen, indicating that those devices were the likely source of infection. Remediation was ultimately achieved through the development and implementation of a device-specific water management program. Manufacturing facilities that use aerosol-generating devices should consider maintaining updated *Legionella* water management programs to prevent *Legionella* bacterial infections.

Although the risk of developing Legionnaires’ disease is generally highest among persons who are >50 years of age, rates in the United States have been increasing for all persons >34 years of age (*1*). A recent study estimated that the actual number of cases might be >1.8–2.7 times what has been previously reported (*1*–*3*). Ongoing challenges such as urbanization, aging populations, racial disparities, and climate change have likely contributed to the increasing number of legionellosis cases occurring globally (*1*,*4*,*5*).

The genus *Legionella* contains >60 species; however, most legionellosis cases in the United States are caused by *Legionella pneumophila*, particularly serogroup 1. *L. pneumophila* is the causative agent for 90% of cases worldwide, followed by *L. longbeachae* (*6*). *L. pneumophila* sequence type (ST) 36 is highly virulent and a frequent cause of both sporadic disease and clusters of legionellosis in the United States and worldwide (*7*–*9*). The first cluster, which had both clinical and environmental ST36 isolates, was investigated by the US Centers for Disease Control and Prevention (CDC) in 1994 (*7*).

Occupational exposure to *Legionella* spp. is a serious health hazard that has been previously reported in industrial settings (*10*). Exposures have been reported from well-known sources, such as cooling towers, hot tubs, and showers (*7*), and more unique sources, such as devices that aerosolize water at high velocity in industrial settings (*10*,*11*). However, legionellosis in industrial facilities can be acquired by exposure to sources not commonly recognized as a cause of illness (*11*). We report a legionellosis cluster among employees of a manufacturing facility in South Carolina, USA, linked to specific equipment exposure sources. This study was reviewed by CDC, was deemed not research, was conducted consistent with applicable federal law and CDC policy (e.g., 45 C.F.R. part 46.102(l)(2), 21 C.F.R. part 56; 42 U.S.C. §241(d); 5 U.S.C. §552a; 44 U.S.C. §3501 et seq.), and did not require review by human or animal subjects research review boards.

## Materials and Methods

### Epidemiologic Investigation

The South Carolina Department of Health and Environmental Control (DHEC) Division of Acute Disease Epidemiology (DADE) used the South Carolina electronic disease surveillance system to collect epidemiologic data for this study. We identified confirmed and suspected legionellosis cases on the basis of the 2020 Council of State and Territorial Epidemiologists case definitions (*12*).

During September 2022, DADE received reports of 3 *Legionella*-positive urinary antigen tests among patients hospitalized with pneumonia who all worked at the same manufacturing facility in Richland County, South Carolina. DADE informed company management about the cluster of legionellosis cases among facility employees and shared an employee awareness notification letter for distribution to employees. Company management informed employees of the legionellosis cluster and the DHEC investigation through both in-person meetings and virtual and electronic communications. DHEC released a statewide health advisory to healthcare providers that had specific recommendations and a reminder to report positive *Legionella* test results and legionellosis cases to DHEC. Because of the occupational setting of the cluster, DADE requested assistance with the epidemiologic investigation from subject matter experts from CDC, including those from the National Institute for Occupational Safety and Health.

Regional epidemiologists conducted telephone interviews of ill persons to collect epidemiologic data for the 14-day period before symptom onset. All patients linked to this cluster were interviewed by using a standardized epidemiologic questionnaire, which gathered demographic information, clinical manifestations, laboratory results, travel history, and potential exposure to high-risk settings and water sources. Patients were asked questions about job title, job description, job location, frequently visited areas aside from the assigned workplace, and visits to other potential exposure sites. Company management provided information about the building and the water distribution systems that included all water processing equipment. We considered the location’s potable water points of use as possible exposure sites, in addition to the water processing equipment. To estimate disease burden, we classified cases into the following 4 categories: confirmed Legionnaires’ disease, defined as a patient who was at the facility and had a clinically compatible case of severe pneumonia with confirmed laboratory evidence of *Legionella* infection and onset of illness on or after May 2022; probable Legionnaires’ disease, defined as a patient who was at the facility and had a clinically compatible case with no laboratory evidence of infection but with onset of illness on or after May 2022; confirmed Pontiac fever, defined as a patient who was at the facility and had a clinically compatible case of mild respiratory disease (no pneumonia) with confirmed laboratory evidence of *Legionella* infection and onset of illness on or after May 2022; and probable Pontiac fever, defined as a patient who was at the facility and had a clinically compatible case with no laboratory evidence of infection but with onset of illness on or after May 2022.

### Case Finding

DADE asked the facility’s occupational health service to actively identify all persons who had new or recent (previous 3 months) self-reported lower respiratory symptoms or clinically diagnosed pneumonia. Those persons were encouraged to contact their primary care physicians for *Legionella* bacteria assessment by using both culture of lower respiratory secretions and a *Legionella*-specific urinary antigen test. In addition, the lead regional investigators (R.W., V.G.) reached out to symptomatic co-workers who were identified by patients with confirmed legionellosis or the employee health service. DADE also conducted a retrospective search of the South Carolina electronic surveillance system for all legionellosis patients who reported working at the same facility within the previous 12 months, in addition to looking for similar worksite exposures reported by patients living in other regions. The DHEC health advisory and employee communications letter promoted seeking early healthcare and made employees aware that they should be tested for Legionnaires’ disease if any respiratory symptoms developed.

### Environmental Investigation and Sampling

Company management hired a *Legionella* consultant (M.G.) who collected preremediation and postremediation environmental samples and sent them to an Environmental *Legionella* Isolation Techniques Evaluation Program member laboratory. The laboratory conducted serial *Legionella* bacteria testing of potable and nonpotable water sources. In preremediation environmental samples, *L. pneumophila* and *Legionella* spp. were detected by using qualitative PCR. Results were reported as detected or not detected. Preremediation and postremediation environmental samples were also collected and tested by using traditional culture (spread plate); those results were reported as colony forming units per volume. The environmental samples were from traditional potential exposure sources, such as showerheads, sinks, and cooling towers, and multiple unique sources, such as floor scrubbers and water jet cutters.

### *Legionella* Bacteria Environmental Risk Assessment

After DADE notified the company of the cluster, company management engaged a consultant with *Legionella* bacteria expertise. They used the CDC’s *Legionella* Environmental Assessment Form to collect information about the water supply, water system design, and potential sources of exposure (*13*). They then used that information to create a water use inventory with detailed information and descriptions for 42 devices, including plumbing and bathroom fixtures, throughout the facility. A map of the facility that indicated where patients routinely worked within the plant was also provided. DHEC staff and CDC subject matter experts conducted a site visit to collect more information about the workplace, including an overview of the cleaning and industrial processes that create aerosolized water, the usual work locations of persons who became ill, and potential areas of stagnation in the facility plumbing system.

## Results

We detected a statewide increase in the number of patients with legionellosis in 2022 compared with previous years; the number (n = 99) was higher in 2022 than the numbers reported during the same time in 2021 (n = 73), 2020 (n = 46), and 2019 (n = 55). We also observed an increase in the number of patients with legionellosis in Richland County (n = 29); fewer than 5 cases in that county were reported during the same period in 2019, 2020, and 2021. In Richland County in 2022, 76% (n = 22) of patients with legionellosis were employees at the facility and were linked to the cluster. No reports of legionellosis were found among residents of the surrounding community, visitors to the facility, or contractors.

### Demographic Characteristics

**Figure 1:**
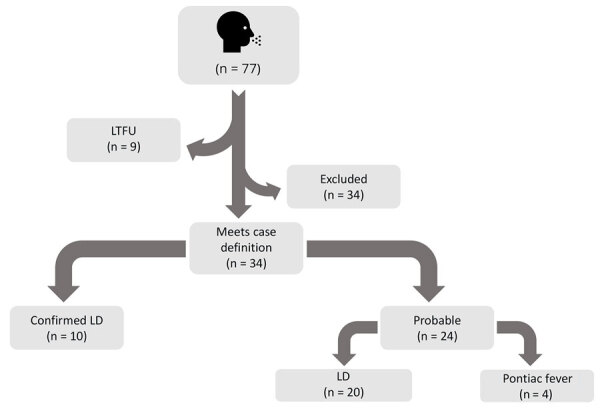
Classification of cases associated with a cluster of legionellosis in a manufacturing facility in South Carolina, USA, 2022. LTFU was defined as failure to reach a patient after 3 attempts within 1 week. Cases were excluded if either the case definition criteria were not met or if the patient had a clinically compatible illness and documentation of an alternative etiology or positive test for COVID-19 or influenza. LD, Legionnaires’ disease; LTFU, lost to follow-up.

A total of 77 workers and other staff at the facility were investigated (Figure 1). Of those persons, 9 were lost to follow-up (defined as failure to reach a patient after 3 attempts within 1 week), 34 were excluded because they either did not meet the case definition or had documentation of an alternative etiology in their medical records, and 34 met the case definition for legionellosis. The 34 patients who met the case definition were classified further; 10 had confirmed Legionnaires’ disease, 20 had probable Legionnaires’ disease, and 4 had probable Pontiac fever (Table 1). Most employees at the facility worked first shift (n = 817 [58.1%]), followed by second shift (n = 460 [32.7%]) and third shift (n = 130 [9.2%]); employees from different shifts had the same work responsibilities. The overall attack rate among employees was 2.4%. The attack rate was 2.1% during the first shift, 1% during the second shift, and 3.8% during the third shift.

**Table 1:** Characteristics of patients with legionellosis associated with a manufacturing facility, South Carolina, USA, May 16, 2022–November 30, 2022*

Characteristics	Case classification, no. (%)			
	Confirmed LD	Probable LD	Probable Pontiac fever	Total
No. cases	10 (29.4)	20 (58.8)	4 (11.8)	34 (100)
Patient sex				
F	3 (27.3)	5 (45.5)	3 (27.3)	11 (32.4)
M	7 (30.4)	15 (65.2)	1 (4.3)	23 (67.6)
Age group, y				
18–49	8 (29.6)	15 (55.6)	4 (14.8)	27 (79.4)
50–64	2 (28.6)	5 (71.4)	0	7 (20.6)
Work shift				
First	4 (23.5)	11 (64.7)	2 (11.8)	17 (50)
Second	2 (50)	2 (50)	0	4 (11.8)
Third	1 (20)	3 (60)	1 (20)	5 (14.7)
Unknown	3(37.5)	4(0.5)	1(12.5)	8 (23.5)
Outcome				
Died	1 (50)	1 (50)	0	2 (5.9)
Hospitalized	9 (60)	6 (40)	0	15 (44.1)
Symptoms				
Cough	10 (31.3)	18 (56.3)	4 (12.5)	32 (94.1)
Fever	9 (31)	18 (62.1)	2 (6.9)	29 (85.3)
Underlying conditions†	3 (37.5)	4 (50)	1 (12.5)	8 (23.5)
Areas of exposure‡				
Break room 1	1 (10)	6 (60)	3 (30)	10 (29.4)
Break room 2	1 (25)	3 (75)	0	4 (11.8)
Break room 3	2 (33.3)	4 (66.7)	0	6 (17.7)
Break room 4	3 (33.3)	5 (55.6)	1 (11.1)	9 (26.5)
Break room 5	3 (60)	2 (40)	0	5 (14.7)
Chiller§	1 (100)	0	0	1 (2.9)
Cooling towers	2 (66.7)	1 (33.3)	0	3 (8.8)
Water jet cutters	2 (25)	4 (50)	2 (25)	8 (23.5)
Sprinkler system	1 (100)	0	0	1 (2.9)
Ice bank§	3 (37.5)	5 (62.5)	0	8 (23.5)
Misters	1 (100)	0	0	1 (2.9)
Drinking water fountain	3 (21.4)	9 (64.3)	2 (14.3)	14 (41.2)

*LD, Legionnaires’ disease. †Patients with any of the following conditions: alcohol abuse, cancer, cerebral accident/stroke, chronic diarrhea, chronic liver disease, chronic obstructive pulmonary disease, diabetes mellitus, heart disease, blood cancer, immunosuppressive condition, drug use, organ transplant, chronic renal failure, sickle cell anemia, or systemic lupus erythematosus. ‡Self-reported frequently visited areas aside from the assigned work station. §Component of the comfort cooling system at the facility that does not aerosolize water.

**Figure 2:**
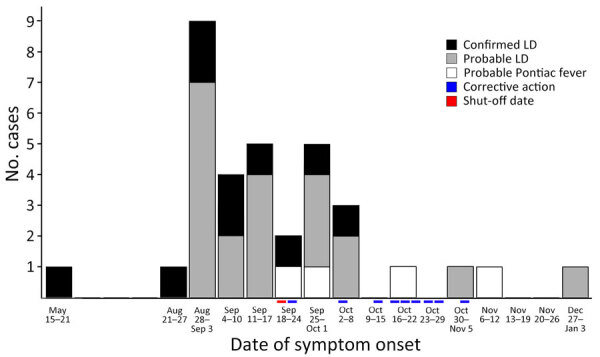
Epidemic curve of reported cases of legionellosis associated with a manufacturing facility and timeline of corrective actions for potential exposure sources, South Carolina, USA, 2022. Of 34 total cases, 10 were confirmed LD cases, 20 probable LD cases, and 4 probable Pontiac fever cases. Red bar on x axis indicates when the cooling towers, water jet cutters, the chiller, and floor scrubbers were all shut off on September 18, 2022. The floor scrubbers were remediated on October 3, 2022. Blue bars on the x axis indicate remedial treatment of remaining water-processing devices on September 22; October 3, 14, 16, 17, 21, 27, and 28; and November 1, 2022. Date of death for 1 patient with probable LD was used as the illness onset date because we were unable to obtain a symptom onset date. LD, Legionnaires’ disease.

**Figure 3:**
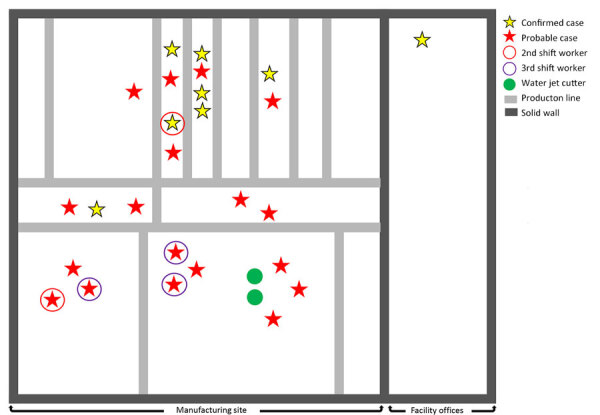
Usual work location of employees with confirmed and probable legionellosis according to shift and proximity to water jet cutters at a manufacturing facility in South Carolina, USA, 2022. Stars without circles indicate first shift workers. Facility is ≈1 × 10^6^ square feet with a ceiling height of 40 feet. Rooftop cooling tower is the primary source of cooling for the building, and air is circulated with industrial ceiling fans throughout the facility. Air flow studies were not performed. Patients reported working in various locations during the 14 days before illness onset. Facility is open-air with 1 interior wall separating the office space from the manufacturing side. Gray lines indicate production lines. Work location information was missing for 8 patients because of investigator inability to identify the location on the map, the employee reported moving throughout the facility, or inability to collect the information from either the employee or company management. Floor scrubbers are not shown because they were used throughout the facility.

More patients with legionellosis were male (n = 23 [67.6%]) than female (n = 11 [32.4%], and the median age was 40 years (Table 1). Fifteen patients were hospitalized because of Legionnaires’ disease. The highest proportion of hospitalizations were reported among persons who were 18–49 years of age (n = 11 [73.3%]), male (n = 10 [66.7%]), and worked during the first shift at the facility (n = 7 [46.7%]) (Table 2). Most (n = 10 [66.7%]) hospitalized patients reported illness onset began during September 2022 (Figure 2). The retrospective search for legionellosis cases in the South Carolina surveillance system identified 1 patient who tested positive for *Legionella* and worked at the facility, reported in May 2022. Two fatalities were reported in this cluster. No clustering of cases according to work locations within the facility was identified (Figure 3). The facility did not keep routine records of employee demographic information; therefore, no comparisons with the general population at the facility could be made.

**Table 2:** Characteristics of hospitalized patients with legionellosis associated with a manufacturing facility, South Carolina, USA, May 16, 2022–November 30, 2022*

Characteristics	Case classification, no. (%)			
	Confirmed LD	Probable LD	Probable Pontiac fever	Total
No. cases	9 (60)	6 (40)	0	15 (100)
Patient sex				
Female	3 (60)	2 (40)	0	5 (33.3)
Male	6 (60)	4 (40)	0	10 (66.7)
Age group, y				
18–49	7 (63.6)	4 (36.4)	0	11 (73.3)
50–64	2 (50)	2 (50)	0	4 (26.7)
Work shift				
First	4 (57.1)	3 (42.9)	0	7 (46.7)
Second	1 (50)	1 (50)	0	2 (13.3)
Third	1 (50)	1 (50)	0	2 (13.3)
Unknown	3 (75)	1 (25)	0	4 (26.7)
Outcome				
Died	1 (50)	1 (50)	0	2 (13.3)
Survived	8 (61.5)	5 (38.5)	0	13 (86.7)
Underlying conditions†	3 (75)	1 (25)	0	4 (26.7)

*LD, Legionnaires’ disease. †Patients with any of the following conditions: alcohol abuse, cancer, cerebral accident/stroke, chronic diarrhea, chronic liver disease, chronic obstructive pulmonary disease, diabetes mellitus, heart disease, blood cancer, immunosuppressive condition, drug use, organ transplant, chronic renal failure, sickle cell anemia, or systemic lupus erythematosus.

### Clinical Sampling Results

Twelve urine specimens from workers at the facility who had symptoms consistent with legionellosis were collected by their healthcare providers and were tested by using an *L. pneumophila* serogroup 1 (Lp1) urinary antigen test either at commercial laboratories or by the South Carolina public health laboratory. Urinary antigen tests for 10 of 12 urine specimens were *L. pneumophila*–positive, indicating infection was most likely from Lp1. For hospitalized patients, we coordinated with infection preventionists to collect lower respiratory specimens if the patient had received antimicrobial drugs for <7 days. We shipped 1 sputum sample to CDC for further testing and characterization. No isolate was recovered from that specimen; therefore, nested sequence-based typing (SBT) results were generated by using DNA extracted directly from the specimen (*14*,*15*). The culture-negative respiratory specimen tested at CDC was identified as Lp1 ST36. 

### Environmental Sampling Results

The *Legionella* consultant collected 316 samples from different potable and nonpotable water sources during September 2022–February 2023. Of those samples, 82 were tested in September 2022 by using qualitative PCR specific for *Legionella* bacteria. *Legionella* spp. and *L. pneumophila* were detected in 37 samples. In addition, 234 samples were tested by using a culture method to isolate *L. pneumophila* and non–*L.* *pneumophila Legionella* spp. All *L. pneumophila* isolates were characterized further to determine if they were serogroup 1. Lp1 was isolated from 8 different samples collected from water jet cutters 1 and 2 (Figure 3) and a floor scrubber in September and October 2022. No *Legionella* spp. were isolated from samples collected from other sources. *L. pneumophila* serogroups 2–15 were isolated from 1 sample collected from a water jet cutter in November 2022. All subsequent samples were negative for *Legionella* bacteria. 

Nine *Legionella* isolates recovered from environmental samples collected at the facility in September 2022 were submitted to CDC for further characterization. CDC performed *Legionella* multiplex real-time PCR on the presumptive *Legionella* isolates and detected Lp1. CDC generated a complete SBT profile for the isolates and identified the environmental isolates as ST36, the same ST as the clinical respiratory specimen.

### Environmental Assessment

The facility provided the following list of records that they maintained: cooling system maintenance records, facility and water inventory maps, *Legionella* Environmental Assessment Form, preventive and maintenance work instructions, safety data sheets, water management plan, and water system inventory. Two water jet cutters were present in the facility and were used on all shifts. The machinery works by combining garnet and water from the premise plumbing system under high pressure to cut parts from sheet metal. The garnet/water mixture is pumped through a circulation loop where the garnet is separated and water is returned to a catch basin. The plant operators had previously determined the basin temperature to be 35°C–40°C during use. The cutting piece and water catch basin were open to the environment, permitting aerosols and water spillage onto the surrounding area.

Floor scrubbers that capture water, sediment, and other particulates throughout the facility were used during all shifts. The manually operated machines sprayed water and detergent on the floor, where the mixture was then spread by large circular brushes and vacuumed into a collection tank. Spray from the machines and the scrubbing motion of the brushes are capable of producing aerosols that could spread *Legionella* bacteria. Although the facility has 5 floor scrubbers, only 1 tested positive for *Legionella* bacteria during this investigation. The area around the water jet cutters was frequently cleaned by the floor scrubbers to remove standing water on the floor resulting from overspray and overflow from the machinery. 

### Environmental Control Measures

Company management shut down both water jet cutters and discontinued use of all floor scrubbers after receiving the initial *Legionella* sampling results. The water jet cutters and floor scrubbers were turned off on September 18, 2022. The water jet cutters were returned to service on November 16, 2022. Although we provided the facility with recommendations on how to safely return the floor scrubbers to service on October 20, 2022, the facility management decided against putting them back in service and used backup scrubbers instead. In response to the legionellosis cluster, the water jet cutter control plan consisted of mechanical preventive measures, such as inspection, cleaning, maintenance, and filter change. The company developed chemical prevention measures, including biocide feed, water quality tests, pump inspections, and the use of slow dissolving tablets of the wide-spectrum biocide, 2,2-dibromo-3-nitrilopropionamide (92%–98%), which can achieve a 4-log reduction in *Legionella* bacteria concentrations when used in appropriate concentrations and causes less corrosion than chlorine (*16*).

Floor scrubber remediation measures consisted of regular mechanical preventive maintenance and disinfection, which included adding chlorine bleach to the recovery tank and filling the tank with clean water. Postremediation samples collected from both the water jet cutters and the floor scrubber were negative for *Legionella* spp.

## Discussion

The legionellosis cluster consisted of 10 confirmed Legionnaires’ disease cases, 20 probable Legionnaires’ disease cases, and 4 probable Pontiac fever cases. The population of workers in the facility were a younger demographic group than is typically associated with Legionnaires’ disease; the median age among the patients was 40 years, whereas the median age of patients with reported Legionnaires’ disease in the United States is 62 years (*1*). The attack rate might be influenced by the shift type. Employees who worked the third shift reported the highest attack rate of 3.8%, which might be caused by a longer exposure time because those employees do not usually leave the building at night. We also observed that patients who reported working on the third shift tended to cluster near to water jet cutters (Figure 3). Although the highest number of patients with legionellosis worked during the first shift, the attack rate was 2.1%; the lower attack rate might have been influenced by the higher proportion of employees who worked in the office in a separated part of the building or worked in other capacities outside the manufacturing part of the building. Results from environmental sampling found 2 water jet cutters and 1 floor scrubber were positive for *Legionella* spp., indicating that those machines were the likely source of the outbreak.

The investigation was initially challenging because a clear exposure pattern did not exist between worksites, devices that aerosolize water, and infection (Table 1). For example, not all patients reported exposures in the same areas within the facility, making it difficult to elucidate patterns of exposure and link infections with specific areas or devices. Also, DHEC staff noted that facility employees did not consistently use the same description to identify sources of water within the facility. However, this lack of a pattern suggested that the source had to be capable of causing widespread exposures.

A visit to the facility by DHEC staff and CDC subject matter experts provided key insights into the environmental and occupational factors that resulted in a relatively large legionellosis cluster within a short period. Any device filled with tap water can grow *Legionella* bacteria (*17*). The main sources of exposure uncovered in this investigation were the water jet cutters and the floor scrubber. The water jet cutters contained reservoirs of water that could reach temperatures of 35°C–40°C, which is ideal for *Legionella* bacterial growth. The water jet cutters also produced substantial aerosols during use that had the potential to travel through the open-floor facility. Remediation of the water jet cutter contamination was complicated by the absence of existing water management recommendations from the equipment manufacturer. Because of vulnerable components in the devices, such as the cutting head that is susceptible to corrosion, chemicals added to the water reservoirs had the potential to damage the equipment.

The use of floor scrubbers during all 3 shifts might have also caused the spread of aerosolized bacteria throughout the facility. A potential biofilm in the cistern of the floor scrubber might have been a contributing factor to *Legionella* bacterial growth and, hence, seeding throughout the facility. Regular maintenance and disinfection of those devices should be prioritized, and individual floor scrubbers should be dedicated to designated areas (i.e., floor scrubbers used near the water jet cutters should not be used in other areas).

The initial corrective and remediation activities started during the second half of September 2022. Legionellosis cases continued to be reported through late November 2022, which might have occurred because of challenges in remediation process implementation that led to sporadic positive identification. However, the epidemiologic curve indicated the number of cases was decreasing after remediation activity, and the last confirmed Legionnaires’ disease cases were reported ≈3 weeks after the initial remediation attempt (Figure 2). During the investigation, the facility identified and tested multiple potable and nonpotable water sources that could pose a potential risk for *Legionella* bacteria exposure. This serial environmental testing of devices that can aerosolize water indicated a second source of exposure was unlikely within the facility. Furthermore, despite the high number of reported cases within the facility, we did not find any other commonalities outside the workplace or identify additional cases from the surrounding communities, leading us to exclude the possibility of an outside source of infection. Although we implemented stricter inclusion criteria for probable legionellosis cases during the influenza season—requiring negative influenza and COVID-19 laboratory test results—the possibility of false positives existed. This possibility might explain the ongoing cases that were reported for several months after the initial remediation attempt and ≈1 month after the last corrective action.

Early reporting of the legionellosis cluster and timely identification of the common occupational exposure among patients were key to limiting the duration of this outbreak. Collaboration with the company’s facility management played a major role in identifying cases and in mitigation to prevent further exposures. Company management distributed notifications to their employees and involved their occupational health service staff to help identify any potential missed cases and ensure that employees sought treatment, if they experienced symptoms. This communication effort combined with prompt reporting to DHEC helped identify cases and potential sources of exposure.

The first limitation of our study is that only 1 lower respiratory sputum specimen was obtained. We did not recover an isolate from that clinical specimen; thus, a higher resolution method of characterization, such as whole-genome sequencing, was not performed on the environmental isolates. Because of the prevalence of *Legionella* ST36 strains, a typing method with more discriminatory power, such as whole-genome multilocus sequence typing, might be required to make inferences about the relatedness of ST36-typed isolates. Finally, the lack of testing for patients with probable legionellosis cases might have resulted in an overestimate if some of those patients did not have Legionnaires’ disease.

In conclusion, clusters of *Legionella* bacterial infections in workplace settings are known to occur and are often associated with cooling towers; however, reports of *Legionella* clusters have been linked to cleaning devices (*7*,*18*). Our experience highlights the need for public health authorities to consider nontypical sources of *Legionella* exposure when investigating legionellosis cases and clusters at manufacturing facilities. It is also critical that owners and operators of water-processing equipment evaluate the risks for legionellosis associated with their use (*10*). Understanding the factors that contribute to the growth and transmission of *Legionella* bacteria is pivotal for effective prevention and control strategies. Manufacturing facilities that use aerosol-generating devices should consider maintaining updated *Legionella* water management programs that specify when, where, and how control measures should be applied to prevent legionellosis cases and clusters.
